# Efficacy of Endoscopic Intervention plus Growth Inhibitor and Patient Self-Management in the Treatment of Esophagogastric Variceal Bleeding in Cirrhosis

**DOI:** 10.1155/2022/6837791

**Published:** 2022-06-15

**Authors:** Zhaoyun Yang, Yizhen Wang, Qin Yu, Shouli Wang, Derun Kong

**Affiliations:** ^1^Department of Gastroenterology, The First Affiliated Hospital of Anhui Medical University, Hefei, Anhui, China; ^2^Digestive Endoscopy Center, The First Affiliated Hospital of Anhui Medical University, Hefei, Anhui, China

## Abstract

**Objective:**

To assess the efficacy of endoscopic intervention plus growth inhibitor and patient self-management in the treatment of esophagogastric variceal bleeding.

**Methods:**

Between January 2019 and December 2021, 60 patients with esophagogastric variceal bleeding treated in our hospital were assessed for eligibility and randomly recruited. They were concurrently and randomly assigned at a ratio of 1 : 1 to receive either endoscopic intervention plus growth inhibitor (control group) or endoscopic intervention plus growth inhibitor and patient self-management (observation group). The endpoint is clinical efficacy.

**Results:**

All eligible patients showed a similar time of hemostasis, success rate of hemostasis, rebleeding rate, and disappearance rate of varicose veins (*P* > 0.05). Endoscopic intervention plus growth inhibitor and patient self-management were associated with a lower incidence of complication (6.67%, including 1 (3.34%) case of ulcer and 1 (3.34%) case of fever) than endoscopic intervention plus growth inhibitor (26.67%, including 3 (10.00%) cases of ulcer, 2 (6.67%) cases of retrosternal pain, and 3 (10.00%) cases of fever) (*P* < 0.05). Patients in the observation group had significantly higher life satisfaction scores (25.17 ± 4.28 and 23.68 ± 5.17) than those in the control group (22.13 ± 2.24 and 18.12 ± 3.28) (*P* < 0.05). A decrease in life satisfaction scores was observed at 6 months after treatment, and the patients given patient self-management showed a higher satisfaction (*P* < 0.05).

**Conclusion:**

Endoscopic intervention plus growth inhibitor and patient self-management yielded remarkable clinical efficacy in the treatment of esophagogastric variceal bleeding as it reduces the incidence of complication and enhances the life satisfaction of patients, and so it is worthy of clinical promotion.

## 1. Introduction

Esophagogastric fundic varices in cirrhosis [1] are one of the main clinical manifestations of portal hypertension and are a common cause of upper gastrointestinal bleeding, which is mainly triggered by liver cirrhosis [2, 3]. Esophageal varices are present in 12%–85% of cirrhosis cases, and about 50% (41%–80%) of gastrointestinal bleeding in patients with portal hypertension is attributed to rupture of varices, while the rest of cases are elicited by gastric mucosal erosion, inflammation, or ulceration. Esophagogastric fundic varices are prone to rupture and bleeding due to elevated portal pressure. Factors such as increased venous reflux blood flow due to negative thoracic pressure, erosion of the esophageal mucosa by acidic reflux in the stomach, and injury due to coarse and hard food or alcohol consumption are causes of rupture and bleeding [4], resulting in complications in patients with cirrhotic portal hypertension. Esophagogastric variceal bleeding is a complication of liver cirrhosis with an incidence of 5–15%, and the rebleeding and mortality rates are as high as 50% and 30%, respectively [5], posing a major threat to the life and health of patients. Therefore, active and effective therapies are crucial to control and prevent the initial bleeding and rebleeding in patients with esophagogastric fundic varices. In recent years, pharmacological treatment in the clinical treatment of esophageal variceal bleeding [6] has obtained good hemostatic effects without little effect on the patient's hemodynamic changes or with other significant adverse effects; however, this method is unavailable for rupture and bleeding of large vessels. Endoscopy combined with drug therapy is a method promoted for clinical use in recent years [7], which provides simple operation and allows direct visualization of the bleeding site and direct hemostatic treatment, which eliminates varices, obtains a good hemostatic effect, and reduces mortality [8, 9]. Nevertheless, the lack of guidance from medical personnel after discharge and the frequent negligence of self-management of patients invariably increase the risk of complications. Growth inhibitor are synthetic tetradecapeptide amino acids with the properties of natural growth inhibitors, which can selectively reduce portal and hepatic blood flow, lower portal pressure and collateral blood flow, and decrease portal collateral circulation blood flow and odd vein blood flow, which may be associated with the significant contraction of the lower esophageal sphincter and the increase of lower esophageal sphincter pressure effect by growth inhibitors. Self-management skills contribute to patients' postoperative recovery and reduce disease recurrence. The recurrence rate of ruptured esophagogastric varices and bleeding in cirrhosis is about 80%, which considerably compromises the quality of life of patients. The quality of life of patients after surgery is severely reduced, and self-management is essential to improve the efficiency of recovery. Accordingly, the present study was conducted to assess the efficacy of endoscopic intervention plus growth inhibitor and patient self-management in the treatment of esophagogastric variceal bleeding. The results are provided in the following sections.

## 2. Materials and Methods

### 2.1. Baseline Data

Between January 2019 and December 2021, 60 patients with esophagogastric variceal bleeding treated in our hospital were assessed for eligibility and randomly recruited. They were concurrently and randomly assigned at a ratio of 1 : 1 to a control group or an observation group. The baseline characteristics of the control group (21 males, 9 females, aged 27–78 years, with a mean age of (52.97 ± 12.17) years, 21 cases of moderate varices, and 9 cases of severe varices) were comparable with those of the observation group (18 males, 12 females, aged 18–74 years, with a mean age of 56.03 ± 13.47 years, 20 cases of moderate varices, and 10 cases of severe varices) (*P* > 0.05) (Table 1). The research was approved by the Ethics Committee of the First Affiliated Hospital of Anhui Medical University, No. 197AH991.

### 2.2. Inclusion and Exclusion Criteria

Inclusion criteria: ① patients met the diagnostic and grading criteria of the 7th edition of Internal Medicine; ② patients were diagnosed with moderate to severe esophagogastric fundic varices by gastroscopy; and ③ the patients and their families were informed of the study and provided written informed consent.

Exclusion criteria: ① patients with coronary heart disease and hypertension; ② patients with serious bleeding tendency; ③ patients with unconsciousness that prevents normal communication; ④ patients with hepatocellular carcinoma or other types of gastrointestinal tumors; ⑤ patients with a history of liver, spleen, or portal vein surgery; ⑥ patients with the use of relevant myelosuppressive or promotive drugs before enrollment; ⑦ patients with coagulation disorders or other hematologic disorders; or ⑧ patients with a history of upper gastrointestinal bleeding 14 days before enrollment.

### 2.3. Methods

All patients were given symptomatic basic treatment such as acid suppression, rehydration, and hemostasis [7], and endoscopic intervention plus growth inhibitor was administered as follows: A suitable injection site (usually 1 cm around the ruptured varicose vein or the most bulged part of the varices) was identified endoscopically, and the site was confirmed using the anterior segment of the extra-needle tube before injection to confirm the varicose vein. The recommended usage of the guideline: the first dose of somatostatin is 250 *μ*g, supplemented intravenously, followed by continuous pumping of 250–500 *μ*g/h. The first dose of octreotide is 50 *μ*g, supplemented intravenously, followed by continuous infusion of 25–50 *μ*g/h. The patients' vital signs and physiological indexes were measured, with cardiac monitoring, and the patients were in absolute bed rest. The patients in the observation group were additionally given self-management interventions. The patient's general condition, occupation, and knowledge levels were evaluated, and individualized treatment protocols were established based on the patient's actual condition by joint discussion among the family, patient, and physician, including discharge guidance, daily intervention, medication intervention, and rehabilitation training. The patients were explained and demonstrated self-management related to the disease and instructed to develop good living habits and self-management skills.

#### 2.3.1. Establishment of Gastroenterology EGVB Management Team

An EGVB management team, comprising two gastroenterologists and three senior nurses, was established to develop and implement a self-management programme for EGVB patients. An intervention WeChat group for EGVB patients was established, and members of the intervention group and patients in the intervention group were added to the WeChat group.

#### 2.3.2. Develop a Detailed Intervention Plan

The interventions were developed through consultation with experts, reference to professional codes and literature review, and included as follows: (1) Education of EGVB-related knowledge: in terms of disease-related knowledge and personal protection after operation, specialized medical staff will explain with live cases and animated pictures and texts, with one theme every day so as to improve patients' self-management ability after operation. (2) Precautions such as daily activities for patients with EGVB and reducing movements or exercises that increase abdominal pressure: monitor pulse and blood pressure at a fixed time and at a fixed location. If vomiting of blood, dizziness, black stools, or irritability are present, there is a high probability of bleeding and early medical attention should be sought. (3) Medication guidance for patients with EGVB: patients can be reminded to take their medication as prescribed by Weibo and telephone, and be informed of the importance of regulating their medication so as to improve their compliance and not to reduce or stop their medication without permission. For example, you should be aware of any adverse reactions to medication such as chest tightness and dizziness. At the same time, follow the doctor's order to regularly go to the hospital for review. (4) Dietary guidance for patients with EGVB: according to the relevant guidelines, formulate and issue a dietary guidance record sheet, indicating the temperature, character, suitable food, fast food, and other detailed precautions of the diet, and keep the stool smooth. Members of the intervention team will post the self-management education content for EGVB patients at least once a week in the WeChat group and interact with EGVB patients on the WeChat platform at any time to answer their questions and concerns.

#### 2.3.3. Intervention Time Frequency

We manage the discharge of patients according to the risk group before discharge and the important time points of bleeding prone. Patients were followed up by micromail and telephone once or twice a week for 1 month after discharge, by micromail and telephone every 2 weeks after 2 to 3 months, and by monthly follow-up interventions for 4 to 6 months.

### 2.4. Outcome Measures

① Treatment: the time of hemostasis, success rate of hemostasis, rebleeding rate, and elimination rate of varices were compared between the two groups. Assessment criteria for successful hemostasis: after treatment, gastroscopic examination shows no jet bleeding or bleeding, indicating that active bleeding has stopped, with visible swollen veins under gastroscopy and no symptoms such as vomiting blood or black stool. Rebleeding assessment criteria: symptoms such as vomiting blood and black stool were seen after active bleeding had been stopped for 24 h after treatment, bloody fluid could be withdrawn from the gastric tube, active bleeding could be seen by endoscopic examination, or systolic blood pressure <90 mmHg and hemoglobin drop >20 g/L could be seen by routine examination.
② Complications: the occurrence of complications (ulcer, retrosternal pain, and fever) was recorded to calculate the incidence of complications.
③ Life satisfaction: the life satisfaction scale was used for assessment. The scale contains 5 domains, namely, my life is generally in line with my ideal life situation, I am very satisfied with my life, I am satisfied with my life, so far I have been able to get the things I wish to have in my life, and there is almost nothing I would like to change if I could change my life choices. Patients rated each domain, from completely disagree to completely agree, on a scale of 1–7 points, respectively, with higher scores indicating higher satisfaction.

### 2.5. Statistical Analysis

SPSS22.0 software was used for data analyses. The count data were expressed as *n* (%) and processed using the chi-square test, and the measurement data were expressed as $\bar{x}$ ± *s* and processed using the *t*-test. Differences were considered statistically significant at *P* < 0.05.

## 3. Results

### 3.1. Treatment Outcome

All eligible patients showed a similar time of hemostasis, success rate of hemostasis, rebleeding rate, and disappearance rate of varicose veins (*P* > 0.05) (Table 2).

### 3.2. Complications

Endoscopic intervention plus growth inhibitor and patient self-management were associated with a lower incidence of complication (6.67%, including 1 (3.34%) case of ulcer and 1 (3.34%) case of fever) than endoscopic intervention plus growth inhibitor (26.67%, including 3 (10.00%) cases of ulcer, 1 (3.34%) case of perforation, 2 (6.67%) cases of retrosternal pain, and 3 (10.00%) cases of fever) (*P* < 0.05) (Table 3).

### 3.3. Life Satisfaction

Patients in the observation group had significantly higher life satisfaction scores (25.17 ± 4.28 and 23.68 ± 5.17) than those in the control group (22.13 ± 2.24 and 18.12 ± 3.28) (*P* < 0.05). A decrease in life satisfaction scores was observed at 6 months after treatment, and the patients receiving patient self-management showed a higher satisfaction (*P* < 0.05) (Table 4).

## 4. Discussion

The high incidence, rebleeding rate, and mortality of esophagogastric fundic variceal bleeding constitute a major threat to the life and health of patients [10]. In recent years, pharmacological treatment has been widely used in the clinical management of esophageal variceal bleeding. Growth inhibitors, or growth hormone release-inhibiting hormones [11], are drugs indicated for the treatment of acute gastric ulcer bleeding, bleeding due to erosion and hemorrhagic gastritis, severe acute esophageal variceal bleeding, and acute pancreatitis, as well as for the prevention of postoperative pancreatic complications [12]. It indirectly blocks vasodilation by contracting visceral vascular smooth muscle and inhibiting the secretion and release of transmitters such as glucagon and vasoactive intestinal peptides [13], resulting in a significant decrease in portal venous trunk blood flow velocity and blood flow, which lowers portal venous pressure to achieve hemostasis. It has been reported [14] that growth inhibitors are favored for their high safety and are available for patients with cardiac insufficiency, hypertensive disorders, and dizziness and headache. Endoscopy [15] can enter the body for internal examination through the natural orifices of the body or through small incisions made surgically, and the combination with endoscopy can improve clinical outcomes in patients with esophagogastric fundic variceal bleeding [16]. Endoscopy combined with drug therapy is promoted for clinical use in recent years, which provides simple operation and a clear view of the endoscope to ensure a more accurate endoscopic operation and a higher hemostasis rate [17, 18], which eliminates varicose veins and compensates for the shortcomings of drug treatment to obtain a promising hemostasis outcome and reduce mortality [19]. The lack of guidance from health care professionals and the frequent neglect of self-management after discharge invariably increase the risk of complications. Self-management education is an effective way of disease management in recent years, which emphasizes the key role of patients in disease management to enhance their self-management ability and improve their quality of life.

The results of the present study showed that all eligible patients showed a similar time to hemostasis, the success rate of hemostasis, rebleeding rate, and disappearance rate of varicose veins (*P* > 0.05). The reason is that growth inhibitors can effectively reduce blood flow in the gastrointestinal tract to achieve hemostasis and inhibit the secretion of acid to provide a clear view of the endoscope, ensure more precise operation, and effectively promote platelet aggregation and vasoconstriction, which is consistent with the research results by Fang Shuixiu. In addition, endoscopic intervention plus growth inhibitor and patient self-management were associated with a lower incidence of complication (6.67%, including 1 (3.34%) case of ulcer and 1 (3.34%) case of fever) than endoscopic intervention plus growth inhibitor (26.67%, including 3 (10.00%) cases of ulcer, 2 (6.67%) cases of retrosternal pain, and 3 (10.00%) cases of fever) (*P* < 0.05). Much of this can be attributed to the fact that the endoscopic intervention plus growth inhibitor treatment involves a smaller injection dose with a properly controlled injection rate, with a lower incidence of adverse reactions, little impact on the patients' systemic hemodynamics, and high patient tolerability; self-management facilitates the patients' postoperative recovery and reduces disease recurrence. Moreover, patients in the observation group had significantly higher life satisfaction scores (25.17 ± 4.28 and 23.68 ± 5.17) than those in the control group (22.13 ± 2.24 and 18.12 ± 3.28) (*P* < 0.05). A decrease in life satisfaction scores was observed at 6 months after treatment, and patient self-management mitigated the reduction of the scores (*P* < 0.05), indicating that endoscopic intervention plus growth inhibitors and patient self-management is effective in improving life satisfaction. Self-management is a new type of patient-centered intervention that enhances patients' self-management ability for their diseases and improves their quality of life to ensure favorable treatment efficacy. Targeted interventions to promote patients' knowledge of disease-related knowledge, medication, diet, and psychological regulation, as well as targeted and personalized health education based on patients' actual conditions, can significantly boost their recovery and achieve an enhanced quality of life, which is consistent with the results of the previous research [20].

To sum up, endoscopic intervention plus growth inhibitor and patient self-management yielded remarkable clinical efficacy in the treatment of esophagogastric variceal bleeding as it facilitates hemostasis, avoids rebleeding and variceal elimination, reduces the incidence of complication, and enhances the life satisfaction of patients, so it is worthy of clinical promotion.

## Figures and Tables

**Table 1:** Comparison of baseline data ($\bar{x}$ ± *s*).

Groups	*n*	Gender		Age		Varices	
		Male	Female	Range	Mean age	Moderate	Severe
Control group	30	21	9	27–78	52.97 ± 12.17	21	9
Observation group	30	18	12	18–74	56.03 ± 13.47	20	10
*t*-value	—	—	—	—	0.834	—	—
*P* value	—	—	—	—	0.411	—	—

**Table 2:** Comparison of treatment outcomes ($\bar{x}$ ± *s*, %).

Groups	*N*	Time to hemostasis (h)	Success rate of hemostasis	Rebleeding rate	Disappearance rate of varicose veins
Study group	30	11.23 ± 4.12	23 (76.67)	7 (23.34)	13 (43.34)
Observation group	30	11.08 ± 3.68	24 (80.00)	7 (23.34)	14 (46.67)
*t*/x^2^	—	0.149	0.098	0.000	0.067
*P* value	—	0.882	0.754	1.000	0.759

**Table 3:** Comparison of incidence of complication (%).

Groups	*n*	Ulcers	Retrosternal pain	Fever	Incidence
Control group	30	3 (10.00)	2 (6.67)	3 (10.00)	8 (26.67)
Observation group	30	1 (3.34)	0 (0.00)	1 (3.34)	2 (6.67)
x^2^	—	—	—	—	4.32
*P* value	—	—	—	—	0.038

**Table 4:** Comparison of life satisfaction ($\bar{\text{x}}$ ± *s*, %).

Groups	*N*	Scores	
		2 months after intervention	6 months after intervention
Control group	30	22.13 ± 2.24	18.12 ± 3.28
Observation group	30	25.17 ± 4.28	23.68 ± 5.17
*t* value	—	3.447	4.231
*P* value	—	0.001	<0.001

*Note.* ^*∗*^indicates statistically significant differences (*P* < 0.05) in scores comparing different periods in the same group.

## Data Availability

All data generated or analyzed during this study are included in this published article.
